# Are asymmetric metal markings on the cone surface of ceramic femoral heads an indication of entrapped debris?

**DOI:** 10.1186/1475-925X-13-38

**Published:** 2014-04-04

**Authors:** Sebastian Valet, Bernhard Weisse, Jakob Kuebler, Martin Zimmermann, Christian Affolter, Giovanni Pietro Terrasi

**Affiliations:** 1 Empa, Swiss Federal Laboratories for Materials Science and Technology, Mechanical Systems Engineering, Duebendorf CH-8600, Switzerland; 2 Empa, Swiss Federal Laboratories for Materials Science and Technology, High Performance Ceramics, Duebendorf CH-8600, Switzerland; 3 Metoxit AG, Emdwiesenstrasse 6, Thayngen CH-8240, Switzerland

**Keywords:** Total hip replacement, Ceramic femoral ball head, Femoral head fracture, Cone surface, Cleaning procedure, Entrapped debris, Taper fit, Metal marking, Physiological loading, Biological contaminant

## Abstract

**Background:**

The probability of in vivo failure of ceramic hip joint implants is very low (0.004-0.05%). In addition to material flaws and overloading, improper handling during implantation can induce fractures of the ceramic ball head in the long term. Identifying the causes of an in vivo fracture contributes to improved understanding and potentially to further reduction of the fracture probability for patients. Asymmetric metal markings on the cone surface of in vivo ball head fractures have been reported. The question, therefore, is whether asymmetric loading is the sole cause or whether additional factors, specifically contamination entrapped in the taper fit, also contribute or are even the main cause.

**Methods:**

The influence of the asymmetric physiological load configuration on resulting metal markings in the cone surface of an alumina femoral ball head with and without biological contaminants was investigated. Static and cyclic tests on ball heads were carried out in a load configuration of 0° (axisymmetric) and 40° in a physiological environment. The analysis of the metal marking was carried out to gain a better understanding of the processes that contribute to the generation of metal marking. Fractography was carried out to determine the fracture initiation of failed ball heads.

**Results:**

Different types and sizes of residuals entrapped in the conical surface are shown to yield strongly asymmetric metal marking patterns. All heads tested without contaminants exhibited an almost homogenous distribution of residual metal markings around the circumference of the ceramic cone surface at the proximal end of the bore hole. The failure of ball heads that contained entrapped contaminants revealed a common fracture pattern. The site of fracture initiation on two of the failed heads was in the entrance region of the bore hole on the superior half of the head.

**Conclusion:**

Asymmetric metal markings observed on the ball heads tested in this investigation are most probably caused by the presence of contaminants entrapped in the taper fit. Homogenous metal mark distributions around the circumference indicate proper assembly of the ball head without entrapped contaminants. It should, however, be noted that different taper designs may possibly result in different marking patterns.

## Background

Total hip replacement (THR) using modular prostheses constitute a state-of-the-art procedure. The use of ceramic femoral heads is becoming increasingly popular. This is due to the excellent immunological biocompatibility, longevity, corrosion and wear resistance [1]. Lower wear rates reduce the risk of osteolysis and premature implant loosening [2]. Furthermore, the use of CoCrMo-alloys as a ball head material is associated with the risk of dissolution into the ionic form and related medical complications. The market share of ceramic femoral heads is therefore steadily increasing [3].

In vivo failures of ceramic femoral heads are rare but possible [4-6]. One of the most recent studies found in the literature reports ceramic femoral head failure rates in the range of about 0.004-0.05%, depending on the manufacturer [6]. Even though the failure probability is nowadays very low, a revision surgery can result in an increased risk due to infection and in turn significant healthcare costs [7]. According to Burns [8], the cost for revision surgeries of total hip replacements in the U.S. in 2003 was 1.7 billion USD compared to 6.7 billion USD for primary hip replacement surgeries. Therefore, all possible measures should be taken to further decrease the failure rate.

Possible causes for a ball head fracture can range from material imperfections through handling faults and material aging to overloading due to extreme activities or impact [9]. While ceramic ball heads have superior tribological properties, i.e. a low friction coefficient, their fracture toughness is low compared to metallic ball heads. As ceramics are brittle, they are susceptible to catastrophic fracture. The risk of in vivo fracture is strongly affected by taper damages or contamination of the stem cone [10-13]. The presence of contaminants (also called entrapped debris) can lead to a non-uniform load transfer inducing stress concentrations in the head, thus increasing the risk of fracture [10-13]. In this study, we focused on entrapped contaminants as a potential cause for ball head fracture. Other potential causes should, however, not be neglected and also have to be considered in the analysis of in vivo fractured ball heads.

In previous studies, the influence of biological contaminants (bone chips, fat and blood) in the stem-ball interface and of a damaged stem cone on the static fracture load of ceramic hip joint ball heads was investigated [10,11]. The experimental studies showed that relatively small entrapped debris, which may be entrapped during surgery, considerably decrease the static fracture load when tested according to ISO7206-10 [14]. During hip surgery, the cleaning procedure for the cone surfaces is increasingly important due to the trend towards more minimally invasive surgical techniques. Thus, smaller incisions may induce a higher risk of entrapping contaminants on the cone surface due to the lower visibility around the stem.

**Figure 1:**
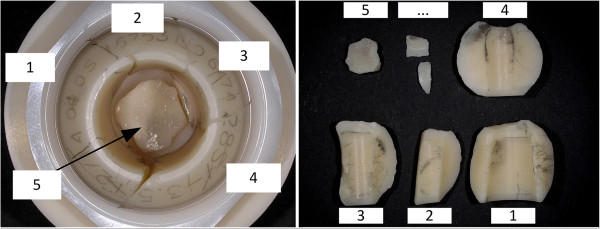
**In vivo fractured femoral head (photograph: Metoxit AG**[19]**).**

Analyses of in vivo fractured ball heads have repeatedly shown asymmetric metal markings on the conical surface of the ceramic fragments [15-18]. Figure 1 shows a typical case of an in vivo fracture of an alumina ceramic head [19]. Inspection of the fractured alumina ball heads typically showed asymmetric distribution of metal marking, resulting, presumably, from the motion in the taper fit interface between the titanium alloy stem and ceramic head prior to fracture (called primary metal marking). Additional metal markings (called secondary metal marking) can be caused by movement between fractured fragments and the stem as they are present on the fragments. Metal markings along fracture edges also appeared on fragments (Figure 1).

The present study aims to clarifying whether asymmetric metal markings could be solely caused by an asymmetric loading of the femoral head or if asymmetric metal markings can be attributed to other causes such as entrapped contaminants. The aim was, therefore, to investigate the influence of in vivo conditions on the metal markings in the conical bore of the ball head in the presence and absence of contaminants. The physical loading condition was simulated by an asymmetric loading configuration.

## Methods

### Test configuration

**Figure 2:**
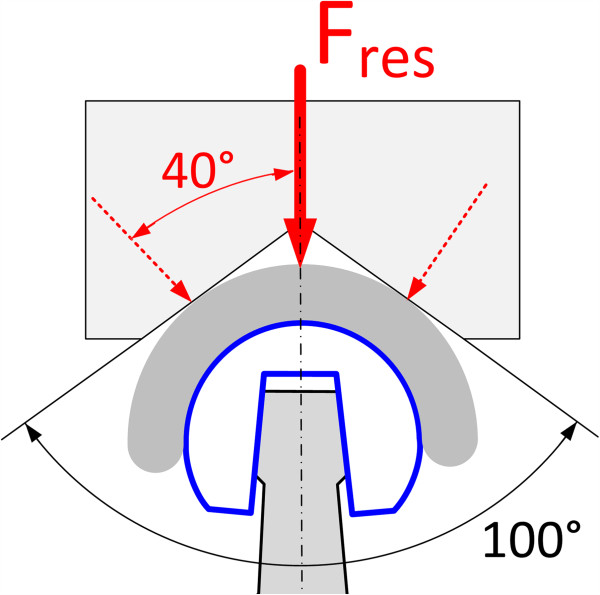
Test arrangement according to ISO 7206–10.

**Figure 3:**
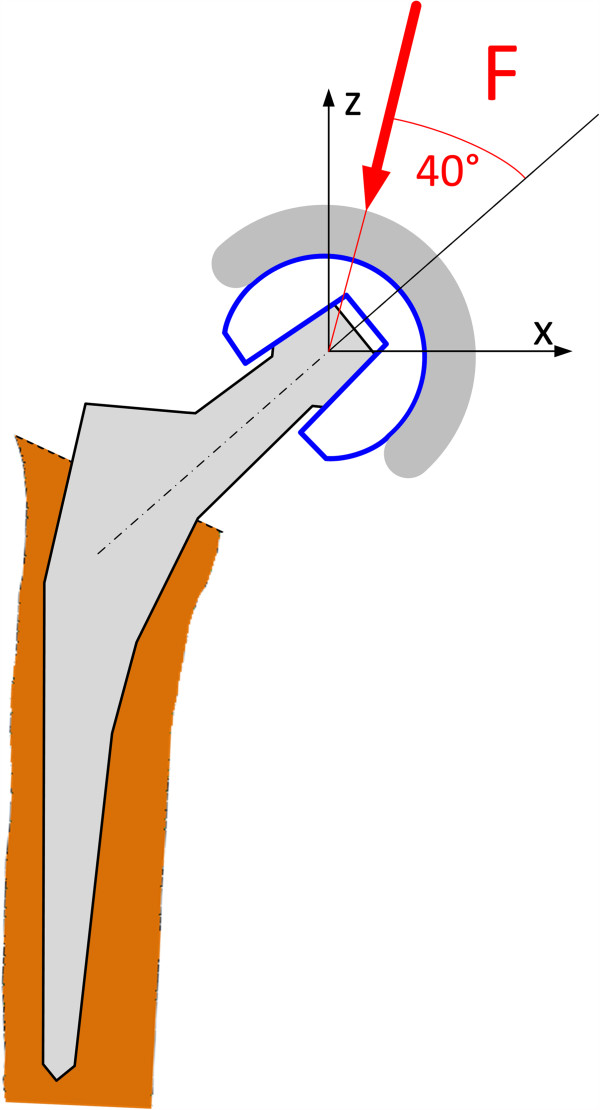
Asymmetric loading of a hip arthroplasty.

The tests that were carried out in this study were subdivided into different stages and are summarized in Table 1. Static ball head assembly tests were carried out with an axisymmetric load configuration. Cyclic tests were carried out with an axisymmetric load configuration acc. to ISO 7206-10 (refer to Figure 2) and an asymmetric load configuration to simulate the physiological loading condition (refer to Figure 3).

**Table 1:** Test configurations

**Type of test**	**Purpose of test**	**Recorded data**
Static axisymmetric test (0°)	Investigation of metal markings for an axial force of 20 and 46 kN.	• Pull-off force
acc. ISO 7206–10 w/o contaminants (Figure 2)		• Metal marking
Cyclic axisymmetric test (0°)	Investigation of metal markings for an axial peak force of 14 kN.	• Pull-off force
		• No. of load cycles
w/ and w/o contaminants (Figure 2)		
		• Metal marking
Cyclic asymmetric test (40°)	Investigation of metal markings for an axial peak force of 4 and 8 kN.	• Pull-off force
w/ and w/o contaminants (Figure 3)		• No. of load cycles
		• Metal marking

**Figure 4:**
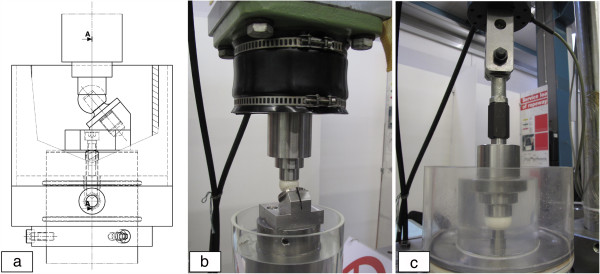
Test arrangement for cyclic tests, a) sketch of 40° setup, b) 40° setup during test in 37°C Ringer’s solution, c) 0° setup during test in 37°C Ringer’s solution.

A test set-up was built and placed in a servo-hydraulic test machine to determine the influence of the physiological load condition on the metal marking characteristic in the conical bore with and without contaminants (Figure 4). In the cyclic configuration, the load was transmitted to the ball head through a spherical stainless steel cup adapter. A rolling table or ball joint was placed between the load cell and the cup adapter to avoid the transmission of transverse forces onto the sample. The load cell (accuracy class 1, EN ISO 7500–2:2004) was attached to the piston of the load cylinder which was fixed to the cross-head of the test machine. The ball head was mounted on a titanium alloy stem (TIAl6V4, Young’s modulus of 122 GPa) that was screwed into a stem holder in an asymmetric (Figure 4-a/b) or axisymmetric configuration (Figure 4-c). The tests were performed in a containment using Ringer’s solution at 37°C. The samples were selected from a manufacturing lot that was intended for testing and research purposes only and not for medical use. A total of 22 samples of Alumina femoral heads (AL BIO-HIP 12/14®, Metoxit, Young’s modulus of 380 GPa) M-type (for medium stem neck length) with a diameter of 28 mm were used in this study. The taper fit was according to the cone system “Eurokonus 12/14”, as this represents a commonly used taper fit system. The cone angle of the stem exhibits a slightly smaller value than in the ceramic femoral head which results in a controlled angular mismatch between the head and stem.

All of the heads used for static and cyclic tests were manually assembled with one impaction stroke using a Polyethylene hammer with a weight of 500 g. The distance between the hammer tip and the prosthesis before applying the impaction was about 30 cm. The assembly force during impaction was not measured.

After performing static and cyclic tests, heads were pulled off using a special setup at a constant displacement rate of 0.5 mm/min. The pull-off force values were recorded to assess the remaining taper fit stability after the test. A statistical correlation between the taper strength and other parameters was not investigated. However, values were compared with data reported in literature.

#### Static assembly tests

The goals of the test were, firstly, to inspect the metal marking that is caused by one hammer stroke as this represents a typical assembly condition for a surgeon, and secondly to investigate the variation in the metal marking depending on the static push-on force.

The pull-off force of three manually impacted heads was determined in a pre-test. They were impacted using the same impaction condition as used for all other ball heads. The mean value and range of the pull-off force was determined from the 3 samples.

Four other ball heads were axially pressed onto the stem taper at a loading rate of 0.5 kN/s according to ISO 7206–10. Prior to all static tests a preload of 2 kN was applied. A 100° cone was used to introduce the load onto the ball head. The assembly force magnitude in the static test was chosen according to the static strength required by the FDA [20]. The FDA specified the requirements on the ball head strength as follows: the average value of a tested group needs to be greater than 46 kN with no single value below 20 kN. In this study, two ball heads were assembled with a push-on force of 20 kN and 46 kN. This represents the most critical conditions that would occur for an assembly up to the required static fracture load. This was expected to cause the strongest possible metal marking on the ceramic cone for loads up to 46 kN. This considers a worst case scenario where the ball head failure load would barely fulfil the strength specified by the FDA. If the actual fracture load of a ball head is greater than the required value of 46 kN even stronger metal marking may be possible.

#### Cyclic loading at 0° and 40°

Cyclic loading tests were carried out in an axisymmetric and asymmetric load configuration using two different test setups (Figure 4). The angle between the load applied onto the ball and the centre line of the taper fit (femoral head-neck axis) was chosen as a realistic mean value to represent asymmetric loading in the daily life activities of a hip joint. The physiological condition that results in asymmetric loading of the taper-fit is schematically depicted in Figure 3.

Experimental studies reported values for the angle between the resulting force acting on the femoral head and the head-neck axis in the range of 28-35° during standing and up to 46° when walking upstairs [21-23].

Based on the reported values, an angle of 40° was chosen for the resultant load as an intermediate value to represent a worst case of asymmetric loading during different activities. Maximum peak forces have been reported to be up to 324% body weight (BW) during walking on flat ground and even up to 870% BW for stumbling [22,24]. In the initial cyclic tests, the lower cyclic load was defined to be 4 kN, representing a load factor of 400% for a motion of an adult with a weight of 100 kg. The type of contaminants that were used in this study remained the same as in a previous study [11]. With these investigations, the intention was to assess the relationship between in vivo load conditions and the metal markings characteristic in the conical bore of the ball head due to the presence of contaminants.

A cyclic compressive load was applied to the sample by using a load peak controller. The load signal was sinusoidal and a load ratio R = 0.1 (F_L_/F_U,_ F_L_: lower compressive load, F_U_: upper compressive load) was applied in all tests. The frequency was set in the range between 6 and 10 Hz. All ball heads were statically preloaded to the mean load F_M_ (between F_U_ and F_L_) with a load rate of 0.5 kN/s before the cyclic load was applied. The test was considered as run-out when the tested sample reached 10 million load cycles without any visible failure.

### Preparation of contaminants

Three contaminant types were considered to represent biological residue that could be entrapped during a THR procedure: (1) fat-free bone chips, (2) soft tissue (fat) and human blood (taken with a blood glucose testing kit) that was dried for one hour before the head was assembled.

**Figure 5:**
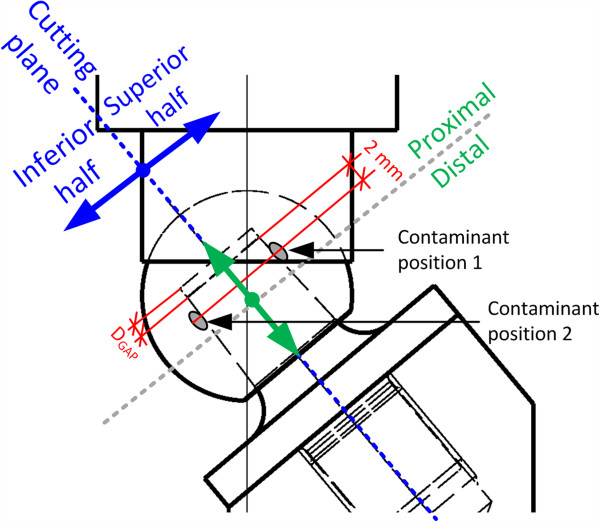
Designation of the contaminant location (directions of interest: superior/inferior and proximal/distal).

**Figure 6:**
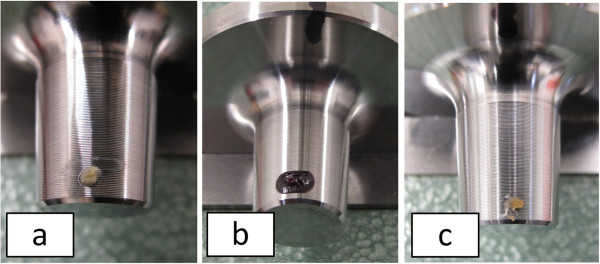
**Application of contaminants on the top position of the stem replacement. (a)** bone chip – 0.8 mg, **(b)**: human blood – 4.7 mg, **(c)**: bovine fat - 1 mg.

Bone chips and fat were glued with a single component, cold-curing cyanoacrylate adhesive (trade name, HBM Z70; commonly used to apply strain gauges). In our previous study [11], the largest reduction in the fracture load was observed when the contaminants were located at the proximal end of the cone surface (Figure 5). In this study, contaminants were again applied at the proximal end of the cone surface, 2 mm from the edge of the proximal end facing in either superior or inferior direction (refer to Figure 5 for orientation, Figure 6 shows contaminants applied on the stem surface). Any position of interest in the conical surface has been designated by two axes in a plane parallel to the coronal plane (A/P). The position was separated into four segments of interest: superior/distal, superior/proximal, inferior/distal, inferior/proximal. The gap between the proximal end of the stem and the bottom of the bore hole was designated as d_GAP_.

The ball head was positioned coaxially on the taper and assembled with one hammer stroke with a Polyethylene head hammer according the instructions for use from the manufacturer [25].

### Analysis of metal marking

**Figure 7:**
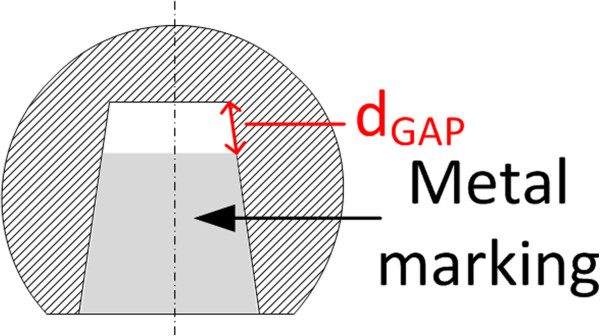
Illustration of measurement position.

The heads were cut in two pieces with a diamond wire saw to inspect the conical ball heads that passed the run-out criterion (refer to Section Cyclic loading at 0° and 40° for the definition of the run-out criterion). The cutting plane was along the head-neck axis of the stem and divided the ball head into a superior and inferior half as indicated in Figure 5. Images of the cross-sectional areas of the head were taken and analysed under repeatable conditions. All images were taken with an optical microscope (Olympus SZX16). The cut side of the sample was positioned parallel to the microscope stage and the camera plane. The axial position of the stem during the test was estimated by measuring the distance between the bottom of the bore hole and the metal marking that corresponds to the proximal end of the stem. The distance was measured along the cone edge in the cross-sectional view and designated as d_GAP_ (Figure 7). The colour of the ceramic material shown in the images does not represent the original colour of the material. Settings were chosen in order to assure good contrast of the metal marking.

## Results

All test results are summarized for the different test configurations that had been investigated throughout the study.

### Static tests according to ISO 7206–10

**Figure 8:**
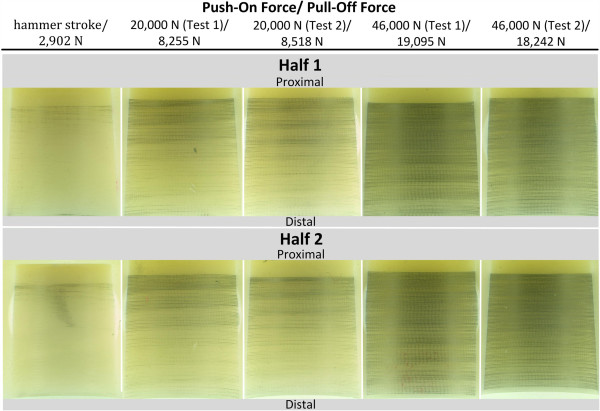
**Metal markings on heads after assembly and pull-off test (for position designation refer to Figure**5**).**

The load–displacement behaviour of all tests showed an almost linear character up to a push-on force of 20 kN and 46 kN. With an increase of the push-on force of 230%, the mean force required to disassemble the heads also increased by 223% (20 kN: n = 2, mean = 8,387 N, range = 263 N; 46 kN: n = 2, mean = 18,669 N, range = 853 N). The ratio between pull-off force and maximum push-on force was in the range of 0.40-0.43 (n = 4, mean value = 0.41, range = 0.03, SD = 0.01). The mean force that was measured during pull-off of ball heads that had been assembled with one hammer stroke was 1,510 N (n = 3, range = 863 N, SD = 293 N). Images of the conical surface of the tested heads are shown in Figure 8. The measured distance d_GAP_ of heads assembled with a load of 20 kN was in a range of 2.3-2.4 mm. On heads assembled with a load of 46 kN, the measured distance d_GAP_ was in a range of 2.1-2.2 mm.

The analysis of the images allowed the following observations to be made:

● **Metal marking characteristics:** The metal marking appeared in a pattern of lines that resulted from manufacturing ridges on the surface of the titanium-taper.

● **Metal marking intensity:** Comparing images of samples that were pushed on the stem with 20 kN and 46 kN, reveals that the intensity of metal markings increased considerably for the higher push-on force.

● **Metal marking distribution along taper axis:** All tested samples revealed a similar distribution of metal marking. A gradient of increasing marking towards the proximal part of the bore hole was present on all samples. However, samples that had been loaded up to 20 kN showed stronger changes in metal marking between the distal and the proximal part of the hole. The marking on samples that were loaded up to 46 kN was more homogenously distributed and showed a smaller intensity change over the conical surface.

● **Metal marking distribution in circumferential direction:** comparing the metal traces of both analyzed halves of the head showed a similar amount and distribution of metal marking on both samples of the same assembly load. The images reveal a homogenous axisymmetric distribution over the surface.

### Cyclic tests

Cyclic tests were carried out on a total number of 18 ball heads. Two samples were loaded in an axisymmetric loading configuration, and 15 samples were tested in an asymmetric load configuration at 40°. The number of load cycles that the samples were exposed to varied depending on the magnitude of the load amplitude, contaminant type and weight.

Asymmetric cyclic tests were mainly performed in order to collect a group of ball head samples that were tested at repeated load conditions and with varying types of contaminant. At an upper compressive load of 4 kN, three ball heads were contaminated with blood and three ball heads were contaminated with bone chips. For both groups, two samples exhibited contaminants at pos. 1 and one sample at pos. 2. Additionally, the ball head contaminated with fat was tested to compare it with the other tested heads. Six samples that contained residuals of blood, bone or fat on the taper cone were exposed to an upper compressive load F_U_ of 4 kN for 10 million load cycles without any signs of failure.

Cyclic tests were carried out with the main objective of investigating how the presence of metal marking is affected by firstly an asymmetric load and secondly by the contamination itself. Therefore, for the latter case, the metal marking on different heads containing contaminants of varying type, size and position was compared under the repeated load conditions. Ball head fractures that were exposed to more than 10^5^ load cycles prior to failure were also considered for the analyses. Such failures are likely to result from fatigue as in the case of in-vivo ball-head failures.

Table 2 shows a summary of all tested run-out samples. With an increased magnitude of peak load and/or weight of contaminant, spontaneous ball fracture occurred in the range between a few load cycles and 3.3 million load cycles. Results from ball heads that failed before the run-out criterion limit are summarized in Table 3. All heads that failed before 10^5^ load cycles were not analysed as these failures were more likely to result from static overloading rather than fatigue mechanisms.

**Table 2:** Run-out samples

		**F**_**U**_**/F**_**L **_**[kN]**	**Weight**	**Contaminant-pos.**	**Cycles**
0°	w/o cont.	14/1.4	-	-	10^7^
		14/1.4	-	-	10^7^
40°	w/o cont.	8/0.8	-	-	10^7^
		8/0.8	-	-	10^7^
		8/0.8	-	-	10^7^
	Blood	4/0.4	0.2 mg	1	10^7^
		4/0.4	4.7 mg	1	10^7^
		4/0.4	5.4 mg	2	10^7^
	Bone	4/0.4	0.7 mg	2	10^7^
		4/0.4	0.8 mg	1	10^7^
	Fat	4/0.4	4.4 mg	1	10^7^

**Table 3:** Ball head failures under cyclic asymmetric loading

	**F**_**U**_**/F**_**L **_**[kN]**	**Weight**	**Contaminant-pos.**	**Cycles**	**Failure/run-out**	**Test-no.**
Blood	8/0.8	7.7 mg	1	< 10^2^	Head fracture	14
Bone	4/0.4	0.7 mg	1	3.28 10^6^	Head fracture	5
	8/0.8	2 mg	1	< 10^2^	Head fracture	3
	8/0.8	0.7 mg	1	< 10^2^	Head fracture	4
	8/0.8	0.7 mg	2	1.25 10^5^	Head fracture	7
Fat	8/0.8	5.4 mg	1	< 10^3^	Head fracture	15
	8/0.8	1.0 mg	1	< 10^2^	Head fracture	17

#### Analysis of 0° loaded heads without contamination

**Figure 9:**
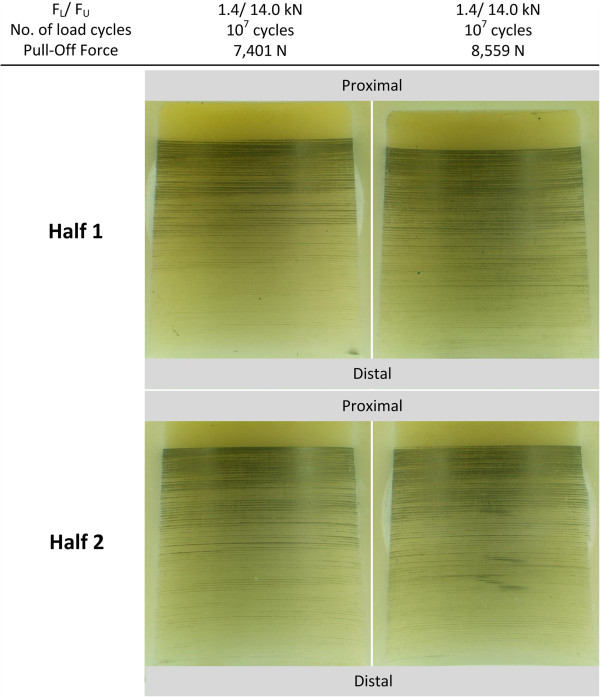
Metal markings on run-out samples cyclically tested under axisymmetric loading.

Both tested ball heads passed the cyclic test at an upper compressive load F_U_ of 14 kN without fracture. No signs of failure were visible on the outside of the head. Both samples were pulled off of the titanium-alloy cone in order to quantify the remaining taper fit stability. A mean pull-off force of 8,215 N was required to pull off the heads after the end of the cyclic tests. This represents a ratio between pull-off and maximum push-on load of 0.35 (n = 2, range: 0.02), which is slightly less than the ratio measured in static assembly tests. The remaining metal markings on the tested ball heads are shown in Figure 9. The measured distance d_GAP_ was in a range of 2.4-2.5 mm.

The following observations can be made for the conical surface:

● **Distribution of metal marking:** an axisymmetric distribution of metallic marking (TiAl6V4) is present along the circumferential direction for both cyclically tested samples. A clear gradient of metal marking is present in the direction of the taper axis. Most metal markings appeared in the proximal area of the cone, whereas slight metal markings are visible at the distal part of the bore hole. The conical surface exhibited similar distribution of metal marking when compared to the samples that had been statically tested.

#### Analysis of 40° loaded heads without contamination

**Figure 10:**
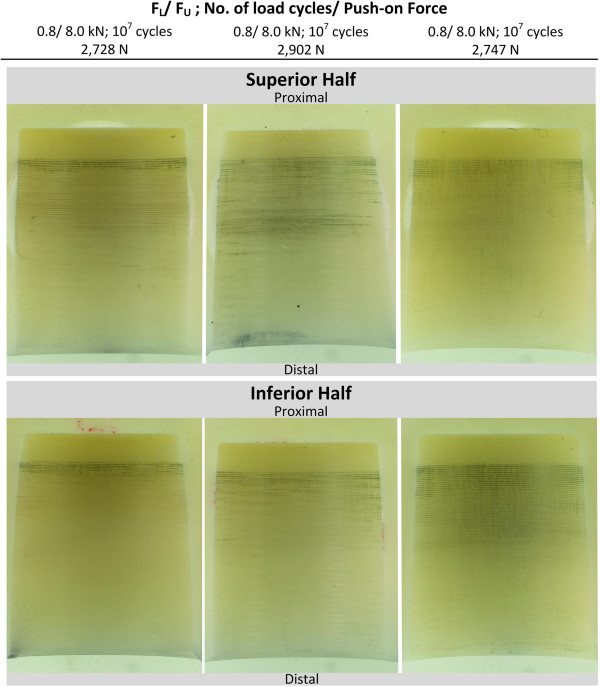
Run-out samples after 40° cyclic test without contamination.

Figure 10 shows the metal marking that occurred under asymmetric loading. Both samples were loaded for 10 million load cycles at a lower compressive cyclic load of 8 kN. A mean force of 2,792 N (n = 3, range: 174 N) was required to pull-off the heads after the cyclic test. The measured distance d_GAP_ was in a range of 2.2-2.5 mm.

The metal markings on the cone surfaces show the following characteristic:

● **Intensity of metal marking:** highest degree of metal marking is present at the proximal end of the bore hole.

● **Distribution of metal marking:** the proximal end of the bore hole of both samples exhibits a homogenous and axisymmetric distribution of metal markings; whereas slightly more marking is present on the superior half of the heads having faced the side of load introduction in the test (refer to images in the top row of Figure 10).

#### Analysis of asymmetric cyclic loaded heads with contaminants

**Figure 11:**
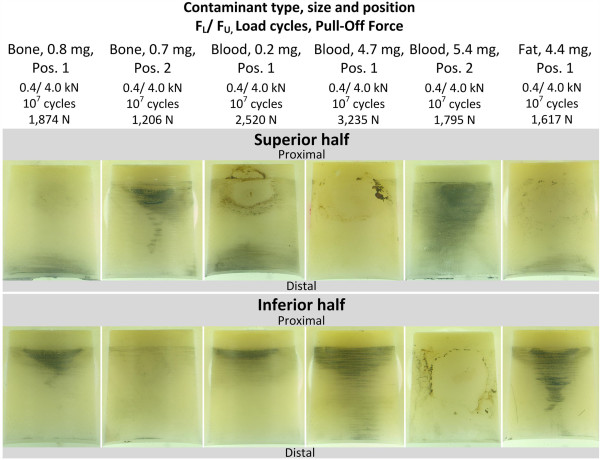
Metal marking on run-out samples containing contaminants tested under asymmetric cyclic loading.

Figure 11 shows metallic marking on heads tested in a 40° configuration that had passed the run-out criterion (10 million load cycles) without failure. The average value of the force required to pull-off the heads from the differently contaminated taper was 2,041 N (n = 6, SD = 721 N, range = 2,029 N). The measured distance d_GAP_ was in a range of 2.5-2.7 mm.

Analysis of the conical surface of all heads with contaminants yields the following observations:

● **Distribution of metal marking in circumferential direction:** A strongly asymmetric distribution of metal markings is present along the circumferential direction.

● **Distribution of metal marking along taper axis:** Two regions of increased metal markings are present. Most intense marking appear in the proximal area at the opposite cone half of the contaminant, whereas slight markings are visible in the distal area on the superior and inferior side of the head.

● **Influence of contaminant position:** If the contaminant was placed on the opposite side of the cone (from superior to inferior side) it did not significantly change the degree and distribution of the resulting metal marking.

## Discussion

The discussion of the aspects addressed in this section may help to better understand the mechanisms that contribute to the generation of metal markings. Several qualitative observations have been made by visually analysing the metal markings. The degree and distribution of metal markings were not quantified. Therefore, the limited amount of quantifiable test data collected in this study, does not allow statistically relevant conclusions to be drawn.

### Static versus cyclic loading

In statically and cyclically tested heads, the strongest metal marking was observed in the contact area between stem and head in the proximal part of the cone surface. This can be attributed to the contact pressure, which is expected to be highest in that region of the taper fit. The stress maximum in this region can be accredited to the angular mismatch between the cone angle of the titanium stem and the femoral head. This causes a non-homogenous press fit with increasing oversize in the proximal direction towards the bottom of the bore hole if the cone angle of the stem is smaller than that of the head.

Metal transferred to the ceramic surface exhibited a negative pattern of manufacturing ridges on the stem in both the statically and cyclically loaded heads. Manufacturing ridges on Morse taper surfaces have a typical feature in order to evenly distribute impaction loads and avoid stress concentrations [2,26] and have an influence on the static strength of a femoral head [26]. This can be explained by the effect of plastic deformation of these manufacturing ridges in the area of an increased contact pressure on the stem-cone surface.

In the tests carried out in this study, the transfer of metal could principally be influenced by different mechanisms ranging from the ball head assembly to the cyclic loading configuration. During assembly, first contact takes place at the proximal end of the stem due to the angular mismatch between the cone angle of the stem and the head [26]. The local peak contact pressure that is induced by the press fit is expected to cause plastic deformation of the stem surface structure. The assembly itself is, therefore, expected to leave metal markings on the ceramic surface. This was confirmed by the analysis of the conical surface of a ball head after it was assembled with one hammer stroke (see Figure 8).

Further metal marking is caused when increasing the push-on load during assembly as the plastic deformation of the ridges continues. The analysis of metal marks from static tests in this study showed that an increase in the assembly load resulted in a more homogenous contact pressure distribution. The comparison of the cone surfaces between heads loaded up to 20 kN and 46 kN indicated stronger and a more homogenous distribution of metal marking for the increased assembly load.

In a Finite Element study by Affolter et al. [27], the contact pressure distribution in the taper interface between a Eurokonus 12/14 and a Al_2_O_3_ head with a size of 28/M was simulated (similar geometry and load as used in this study) for a static load of 20 kN (frictional coefficient: 0.35). The hoop stress along the cone surface was reported to increase towards the proximal part of the bore hole up to a value of about 130 MPa. As a consequence, the contact pressure is also expected to increase in the proximal direction. This is an indication that the increase in metal marking can be related to the increase in contact pressure caused by the angular mismatch.

Another mechanism that contributes to the generation of metal marking may be the micromotion between the head and the stem during cyclic loading. The magnitude of micromotion has been reported to be influenced by factors such as design parameters [17,18] and the load level during a load cycle [28]. The latter study estimated the quantity of micromotion in the taper interface between stem and neck adapter of a bimodular hip prosthesis. The magnitude of micromotion was positively correlated with increasing load levels of a sinusoidal load cycle. Although a different taper fit system was used in that study, an increase in the cyclic load amplitude is also expected to result in an increased degree of micromotion at the taper fit interface in our study. However, it was not within the scope of this study to investigate the influence of micromotion on the metal marking generated on the ceramic cone surface. The quantity of metal marking accumulated over time was, therefore, not determined. The metal marking caused by the pre-loading procedure and the metal marking caused by the cyclic loading were not distinguished.

### Influence of asymmetric loading on metal marking

The area of highest contact pressure exhibited a homogenous distribution of metal markings without interruption in circumferential direction independent of load direction. For ball heads that had been tested under 0° load, metal marking also showed a homogenous axisymmetric distribution. Heads that had been tested in an asymmetric loading configuration did not show a strong asymmetric distribution of metal. This was contrary to the expectation that an asymmetric loading induces an asymmetric stress distribution inside the head [27,29]. Two of the tested heads showed only slightly stronger metal marking in the proximal region of the head (Figure 10). Although the inner cone surface of the ball showed slightly asymmetric metal marking, the area of strongest marking exhibited a homogenous distribution of metal along the circumferential direction. This was contrary to the expectation that an asymmetric stress distribution also results in asymmetric metal marking. One explanation for this is that the asymmetric stress distribution is compensated for by the mechanism of local plastic deformation of the manufacturing ridges on the taper surface.

### Influence of contaminants on metal marking

Different types and sizes of contaminants caused a strong non-axisymmetric distribution of metal marking in all tested heads. Analysis of the samples showed that the presence of a contaminant principally results in strong metallic contact markings concentrated in two regions. This could be observed with good repeatability for all heads where contaminants were entrapped in the taper surface. However, the marking pattern of the metal was influenced by the magnitude of the applied load, as well as the size, type and position of contaminant. One region of concentrated metal markings was located on the opposite side of the contaminant position while the second region of increased metal markings was on the same side as the contaminant but on the distal side of the ball head bore. The concentration of metal markings in two regions can be explained as follows.

The presence of a contaminant introduces an eccentricity and misalignment between the taper axes of the stem and the conical hollow of the ball head. Thus, additional contact pressure on the distal end on one side causes a tipping of the ball, which then creates an increased contact pressure at the proximal end but on the opposite side.

**Figure 12:**
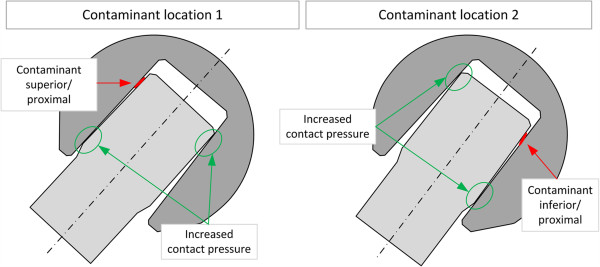
Illustration of regions with increased metal marking on heads containing contaminants for a 40° load configuration (only a qualitative illustration, dimensions in the sketch are not to scale).

Figure 12 qualitatively shows how the taper interface is affected by the presence of a contaminant. The illustrated schematic shows the taper-interface fit in a cross-sectional view along the cone axis and in the plane of the applied test load and contaminant position (Figure 12). Residuals concentrated on one position and side of the stem surface will, if not completely compressed, result in a slight tilt of the stem-cone against the cone of the femoral head. This situation increases local contact stresses in two regions as depicted in the sketch (Figure 12). As metal markings were predominant in the regions of increased contact stresses, it can be proposed that they were caused by the increase in local contact pressure in these regions.

The increase in contact pressure is expected to induce a larger degree of plastic deformation. This can also be accompanied by the micromotion of ridges causing metal markings concentrated in these regions. Furthermore, the presence of contaminants could also have increased the micromotion between the taper stem and the ball head. The influence of bone chips entrapped in the stem-neck interface of a modular hip endoprosthesis on micromotion has been investigated in a study by Jauch et al. [30]. The degree of micromotion has been reported to increase if contaminants are entrapped in the taper fit. The work of Rehmer et al. [31] has shown that the pull-off load is nearly proportionally increasing with raised impact load for different material combinations of a taper fit. The authors further suggested that a minimum peak load of 4 kN should be applied to a Ti-ceramic taper fit to ensure a sufficient taper fixation. An insufficient taper fixation in turn is also likely to result in an increase in micro-motion in the taper fit. Lower levels of impact could therefore also influence the asymmetry of the witness marking. However, further investigation would be required to confirm this presumption.

The position of the contaminant did not influence the principle of asymmetric metal marking distribution. Independent of the position, metal marking was present near the proximal end of the stem on the opposite side of the contaminant as well as in the distal region on the same side of the cone where the contaminant is also present.

### Influence of contaminants on taper disassembly forces

Comparing the forces of the pull-off disassembling procedure carried out on all non-fractured heads allows an evaluation of the remaining pressfit stability of the tested heads. The ratio between pull-off forces and assembly/impaction forces determined in reference [31] for a Ti-ceramic combination of a modular stem-head interface represents a reference value for a press fit without contamination. A correlation between assembly force and pull-off force has been reported as F_pull-off_ = 0.51 F_peak-load_ + 222.1 N for a static push-on assembly and as F_pull-off_ = 0.36 F_peak-load_ + 119.9 N for a single stroke impaction with a rubber-tip. Lavernia et al. [32] investigated the influence of blood/fat entrapped in the taper surface during the assembly procedure and measured lower pull-off forces for contaminated taper-fits than for clean ones. By contrast in our study a significant influence of the contaminants on the remaining press fit stability after 10 million load cycles could not be observed. This may be explained by the degree of contamination that is expected to be much lower as it did not result in failure after 10 million load cycles. However, it should be taken into account that the heads had been exposed to different conditions. The study of Lavernia et al. [32] tested the pull-off force immediately after assembly, whereas the cyclic loading used in this study may also have influenced the resulting pull-off force.

The ratio between pull-off force and maximum compressive load applied to the taper fit over all static and cyclic tests in an axisymmetric configuration carried out in this study varied in a range between 0.40 and 0.61 (n = 6, mean value = 0.47, SD = 0.09/ 19%, range = 0.21/46%).

The ratio between 0° pull-off load and 40° testing load varied between 0.34 and 0.81 for asymmetric loading. A separation into one group of 40° heads tested without contaminants and one group of heads tested with contaminants results in average values of 0.44 (n = 3, SD = 0.01/3%, range = 0.02/6%) and 0.51 (n = 6, SD = 0.18/36%, range = 0.51/99%), respectively. However, the highest ratio values were determined for run-out samples contaminated with blood (n = 3, mean = 0.63, SD = 0.18/29%, range = 0.36/57%) and the lowest for samples contaminated with bone or fat (n = 3, mean = 0.39, SD = 0.08/21%, range = 0.17/43%). The small amount of data points does not allow statistically significant evaluation. However, differences between bone/fat and blood may be explained by a more homogenous contact pressure distribution for samples that had contained blood or fat residuals compared with samples that contained bone chips. It is also interesting to note that Pennock [33], in contrast to Lavernia [32] had also measured increased pull-off forces for heads with bovine serum or water entrapped in the taper cone when compared to heads impacted with a dry and clean cone surface. One explanation may be that a decrease in the frictional resistance resulted in a stronger press-fit.

### Fractographic analysis

**Figure 13:**
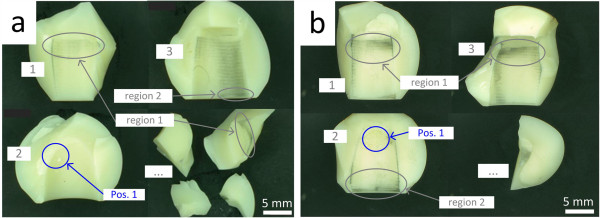
**Fractured ball heads selected for in-depth fractographic analysis. a)** Test no. 15 (<103 cycles) and **b)** Test no. 5 (3.3 106 cycles). “Region 1/2” designates the regions of increased contact pressure for the presence of a contaminant.

**Figure 14:**
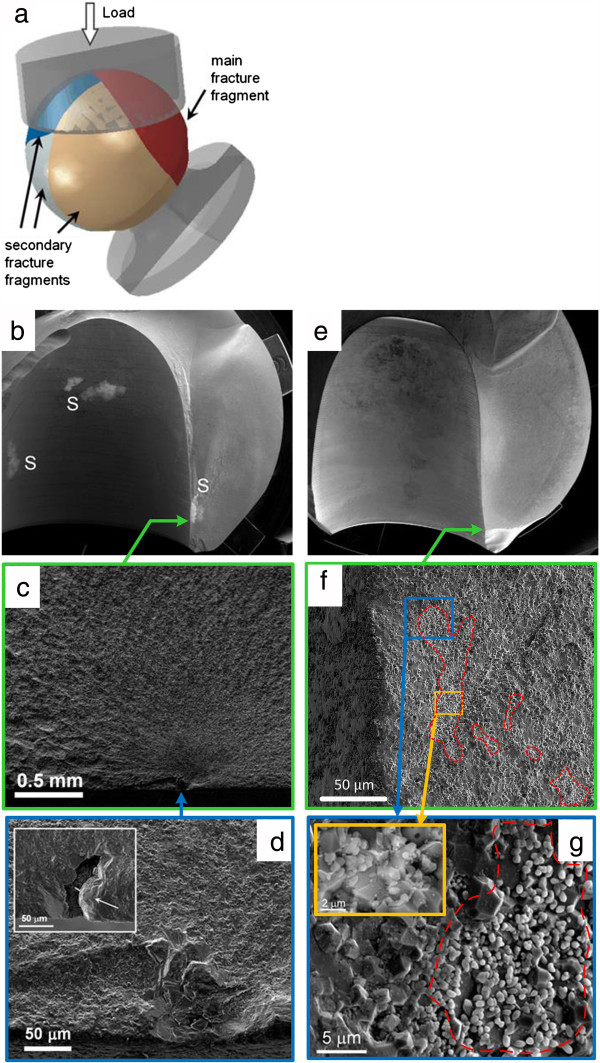
**Fratographic analysis. a)** General fracture behaviour of ball heads failing before reaching 107 fatigue cycles. **b, c, d)** Test Nr. 15 (<103 cycles) and **e, f, g)** Test Nr. 5 (3.28*106 cycles) **b, e)** Main fracture fragment (fish‒eye mode). Arrow marks fracture origin and S salt scales. **c)** Arrow marks fracture origin. **d)** Fracture origin magnified. Insert: opposite fracture surface. Arrow marks a granule, attached to large pore. The granule had not been destroyed during pressing the green body. **f)** Fracture origin magnified (circular line marks area in which porous regions where found). **g)** Magnified area showing a fine grained contamination. Insert: Magnified area BS shown in BSE mode.

Seven of the total 18 femoral heads tested failed before reaching the test stop criterion of 10^7^ cycles. Four of those seven heads (test no. 5, 7, 14 and 15) were selected for a first failure analysis which revealed that the heads had a common failure pattern. Independent of the contamination position (1 or 2) between stem and head bore and as a result of the loading configuration a main fracture fragment with spherical calotte shape was detached from the superior half of the heads (Figure 13a). For a more in-depth failure analysis the femoral heads with the lowest number of load cycles till failure (test no. 15, <10^3^ cycles) and the one with the highest number (test no. 5, 3.28*10^6^ cycles) were selected, Figure 14. Figure 13b and Figure 13e show the main fracture fragments of the two heads with arrows marking the fracture origins. On the main fracture fragment of test no. 15 two white spots could be seen in the bore and one covering the failure origin. The spots originated most likely from the salt in the Ringer-solution in which the tests were conducted at a temperature of 37°C and could be removed easily with lukewarm water. That femoral head failed from a large pore connected to a granule which had not been destroyed during pressing of the ceramic green body, Figure 13c and Figure 13d. The other head (test no. 5) failed from a porous region, Figure 13f. In the porous region loose, slightly necked or aggregated grains with a diameter in the range of 0.5 to 1 μm could be found, Figure 13g. In a back scattered (BS) electron microscope image almost no difference in the grey levels of the grains and the surrounding Al_2_O_3_ ceramic can be seen, insert in Figure 13g. Therefore, the grains might be not properly densified Al_2_O_3_ particles or, more likely fine grained powder from a sintering additive to reduce grain growth, when sintering Al_2_O_3_.

With the failure relevant defect size (depth) in test no. 15 which is, based on the fractographic images, roughly a = 120 μm and the Griffith equation [34] it is possible to estimate that the relevant stress σ at failure had been about in the range of 280 MPa. For test no. 5 it was not possible to estimate a relevant failure size fractographically.

$$K_{\mathrm{lc}}=\sigma \sqrt{a}Y$$

[34] with:

K_Ic_ fracture toughness (typically approx. 4 MPa √m for high purity Al_2_O_3_ ceramic)

Y geometric constant (approx. 1.3 at the surface of a semi-circular crack)

a crack length

Fracture of samples at the beginning of the cyclic testing may be attributed to either static overloading during the increase of the cyclic test load or to low cycle fatigue due to subcritical crack-growth. In either case stress concentration induced by the contaminants are likely to have contributed to the failure. Asymmetric metallic traces found on the fragments of the in-vivo fracture as well as of the ball head fracture under asymmetric test conditions are an indication of an asymmetric contact pressure distribution. This asymmetry can be caused by the presence of contaminants resulting in locally increased contact stresses. In this study, all tested heads containing different types and sizes of contaminants exhibited significant asymmetric distribution of metallic marks on the conical surface. Contaminants such as bone chips, soft tissue or blood entrapped in the taper fit were reported to lower the fracture load in a test according to ISO 7206–10 by up to 90% [9,11]. The types of fracture observed in the failed heads of the present study were similar to the typical bore failure as reported for a static axisymmetric burst test [1].

Therefore, we suppose that the failure of the ball heads was caused by an increase of the maximum stresses in the ball head due to the presence of the contaminants. Additional analysis would be needed to quantify the increase of maximum stress if a contaminant is entrapped in the taper fit. This analysis may be conducted in a separate study.

The distribution of metallic traces transferred to the ceramic cone surface prior to the in-vivo fracture can give some information about the correct assembly of the ceramic ball head onto the stem taper during surgery. A strong asymmetric distribution of metallic traces is likely to have resulted from the presence of contaminants in the conical surface or other defects in the taper fit surface.

## Conclusion

The main goal of this study was to investigate the influence of loading configuration and the presence of contaminants entrapped in the taper fit on the resulting pattern of metal markings on the conical surface of a ball head. Static tests at 20 kN and 46 kN according to ISO 7206–10 as well as cyclic tests in an axisymmetric and asymmetric loading configuration were carried out. Femoral heads that passed 10 million load cycles without failure were disassembled and the pull-off force was measured. All tests were evaluated in terms of remaining metal markings as well as remaining press-fit stability.

On all analysed cone surfaces, metal marking resulting from interface motion between ball head and Ti-alloy were present. Both cases of axisymmetric and asymmetric loading resulted in metal marking with the highest intensity in the taper contact area at the distal end (most inner part) of the bore hole. Metal marks in this area were homogenously distributed around the circumference when no contaminants were present. It can, therefore, be concluded that even under asymmetric loading, the area where most metal markings are generated exhibits, an axisymmetric distribution. This may be explained by the flattening of manufacturing ridges on the stem surface compensating for a non-homogenous contact stress distribution.

The entrapment of even small amounts of contaminants such as blood (0.2 mg, 4.7 mg), bone chips (0.7 mg) or fat (4.4 mg) resulted in an asymmetric generation of metal markings when loaded in an asymmetric direction at an upper compressive load of 4 kN for 10^7^ cycles. An increase in contaminant weight as well as an increase in the testing load resulted in femoral head fracture. The remaining metal markings on the ceramic fragments also exhibited an asymmetric distribution of metal markings. All failed ball heads revealed a common fracture pattern. Independent of the contaminant position, failure of the head with the detachment of a spherical calotte shape fragment from the superior half of the head. The site of fracture initiation on two heads was identified to be in the entrance region of the borehole on the superior side of the head. In this area also an increased metal marking was present which can be explained by an increased contact pressure in that region due to a tilt of the stem against the ceramic cone.

The occurrence of asymmetric metal markings on the cone surfaces of fractured femoral heads can be an indication that either a biological residual was entrapped in the taper fit or the stem cone was damaged prior to fracture. The entrapment of contaminants in the taper fit surface introduces an increased risk of femoral head fracture. Therefore, particular care should be taken during surgeries to assure the cone surfaces of the stem and of the ceramic ball head are cleaned and dried properly before impaction of the femoral head.

It should be noted that the results described in this study are limited to the taper geometry used for all tests in this study (Metoxit 28 M, 12/14 taper, Eurokonus). The use of a different taper design could potentially result in different marking patterns. Unless there is evidence that the presence of contaminants will result in conditions that are less critical compared to the design described in this study, special care should be taken in the cleaning procedure of all taper sizes.

## Abbreviations

SD: Standard deviation; BW: Body weight; FDA: Food and drug administration; A/P: Anterior-posterior direction.

## Competing interests

Metoxit AG and Empa initiated the idea to investigate the issues discussed in this manuscript. The specimens were provided by Metoxit. The study design and the testing concept were developed by Empa. All tests were carried out and evaluated independently by Empa. The study was financially covered by Empa.

## Authors’ contributions

SV planned experimental procedures, prepared test samples, carried out experimental tests, analysed test samples, interpreted and discussed test results as well as drafted and revised the manuscript. BW partly initiated the idea of the study, planned the study design, concept and test procedures as well as prepared samples, carried out experimental tests and helped to draft and revise the manuscript. MZ partly initiated the idea for the study, provided test specimens and helped to draft the manuscript. CHA participated in scientific and technical discussions, helped interpreting test data and helped to draft the manuscript. GPT participated in scientific discussions about the test results and helped drafting the manuscript. JK conducted the failure analysis of the fractured ball heads. All authors read and approved the final manuscript.
